# Statistical Analysis of Individual Participant Data Meta-Analyses: A Comparison of Methods and Recommendations for Practice

**DOI:** 10.1371/journal.pone.0046042

**Published:** 2012-10-03

**Authors:** Gavin B. Stewart, Douglas G. Altman, Lisa M. Askie, Lelia Duley, Mark C. Simmonds, Lesley A. Stewart

**Affiliations:** 1 Centre for Reviews and Dissemination, University of York, York, United Kingdom; 2 Centre for Statistics in Medicine, University of Oxford, Oxford, United Kingdom; 3 NHMRC Clinical Trials Centre, University of Sydney, Sydney, Australia; 4 Nottingham Clinical Trials Unit, University of Nottingham, Nottingham, United Kingdom; Sapienza University of Rome, Italy

## Abstract

**Background:**

Individual participant data (IPD) meta-analyses that obtain “raw” data from studies rather than summary data typically adopt a “two-stage” approach to analysis whereby IPD within trials generate summary measures, which are combined using standard meta-analytical methods. Recently, a range of “one-stage” approaches which combine all individual participant data in a single meta-analysis have been suggested as providing a more powerful and flexible approach. However, they are more complex to implement and require statistical support. This study uses a dataset to compare “two-stage” and “one-stage” models of varying complexity, to ascertain whether results obtained from the approaches differ in a clinically meaningful way.

**Methods and Findings:**

We included data from 24 randomised controlled trials, evaluating antiplatelet agents, for the prevention of pre-eclampsia in pregnancy. We performed two-stage and one-stage IPD meta-analyses to estimate overall treatment effect and to explore potential treatment interactions whereby particular types of women and their babies might benefit differentially from receiving antiplatelets. Two-stage and one-stage approaches gave similar results, showing a benefit of using anti-platelets (Relative risk 0.90, 95% CI 0.84 to 0.97). Neither approach suggested that any particular type of women benefited more or less from antiplatelets. There were no material differences in results between different types of one-stage model.

**Conclusions:**

For these data, two-stage and one-stage approaches to analysis produce similar results. Although one-stage models offer a flexible environment for exploring model structure and are useful where across study patterns relating to types of participant, intervention and outcome mask similar relationships within trials, the additional insights provided by their usage may not outweigh the costs of statistical support for routine application in syntheses of randomised controlled trials. Researchers considering undertaking an IPD meta-analysis should not necessarily be deterred by a perceived need for sophisticated statistical methods when combining information from large randomised trials.

## Introduction

Individual participant data (IPD) systematic review and meta-analysis in which the original “raw” data from each participant in the relevant trials are centrally collected, checked, re-analysed and combined [1], [2], is considered to be a gold standard approach to evidence synthesis. The IPD approach has the potential to minimise publication and reporting biases [3] and to allow detailed data checking and verification. Analysts can re-code covariate, measurement and outcome data to common definitions and carry out appropriate analyses, even where trials failed to do so [4]. A major advantage of IPD analysis over the conventional aggregate data approach is that it allows detailed participant-level exploration of treatment effectiveness in relation to individuals’ characteristics such as age or stage of disease [2], [5].

To date, most IPD analyses have taken a two-stage approach to analysis. In the first stage individual participant data within a trial are analysed to generate trial-level summary statistics (e.g. relative risks). In the second stage these results from each trial are combined across trials using conventional meta-analytical methods [6], [7].The two-stage approach is relatively straightforward to implement, and produces easily interpretable and communicable results for those familiar with meta-analyses of aggregate data.

A “one-stage” approach, by contrast combines all individual participant data in a single meta-analysis based on a regression model stratified by trial (e.g. a logistic regression). In order to incorporate random-effects to allow for heterogeneity, hierarchical or mixed-effect regression models are used [7]–[9]. These models are particularly suitable for investigating how treatment effects vary between individuals or groups [10] and have improved ability to detect differences between groups of participants over two-stage meta-analyses. They also allow separation of group level and individual level relationships; and allow models with different statistical assumptions and structure to be formally compared.

In the two-stage framework associations between treatment and participant characteristics may be investigated by subgroup analysis commonly accompanied by tests of interaction [6], [11], or less commonly by meta-regression [12]. These indirect comparisons present two problems both of which are potentially addressed by the use of one-stage models. First, because they are trial-level comparisons they lack statistical power to detect an interaction when compared to using all the data across trials [13], [14]. One-stage models improve the power to detect treatment by covariate interactions in IPD meta-analyses [15], [16], suggesting that one-stage models may be most useful where trials are few or small, i.e. they have limited power. Second, the association between effect and covariate in a subgroup analysis or meta-regression may consist of a mixture of within-trial relationships and across-trial relationships resulting in the potential for aggregation or ecological bias [13], [17], [18] (definition and explanation provided in supporting information S1, figure S1). Aggregation bias in a two-stage approach may be avoided by estimating interaction parameters separately in each trial and then combining these estimates using conventional meta-analysis [16], [19].

The one-stage approach is flexible, allowing incorporation of both random treatment effects [8] and random-effects on treatment-covariate interaction terms [9]. Multiple patient factors (covariates) may be incorporated in a single model - provided sufficient data are available for all trials. Correlation between covariates and trials can also be explicitly included. Aggregation bias may be avoided by analysing only within-trial relationships between treatment and covariates or by estimating within and across-trial treatment-covariate interactions independently [9]. Different one-stage models may be compared in terms of goodness-of-fit (how well the model explains the data) and complexity, using the Akaike Information Criterion (AIC) [20], providing a means of choosing between multiple models.

The flexibility of the one-stage approach offers multiple approaches to model specification and so increases the potential for data dredging [21]. The relative complexity makes communicating results more difficult. The reduced flexibility of the two-stage approach minimises the chances of data dredging, but implicit assumptions may be inappropriate [22], [23], particularly with heterogeneous data [24]. For instance, over-fitting may occur when the number of studies is (relatively) small or the normality assumption of random-effects is violated [25]. Although one-stage approaches require explicit value judgements about how syntheses could be optimised, they can provide alternative analytical strategies that may either overcome these problems or demonstrate the sensitivity of results to specific model assumptions.

There is currently no consensus or guidance on the appropriateness of the different approaches to analysis of IPD [26]. A recent paper advocated either a two-stage approach to combining within-trial treatment-covariate interactions based on regression or one-stage models [19]. Comparisons of one and two-stage methods based on time-to-event data have suggested that choice between them has limited impact on treatment effects or treatment-covariate interactions, although arguably, one-stage models may provide “deeper insights” into the data [27], [28].

Here we present an empirical comparison of one and two-stage methods for dichotomous outcome data, based on a large individual participant dataset which includes both large and small randomised controlled trials (range 22 to 8016 participants). We compared fixed-effect and random-effects estimates of overall treatment effectiveness and treatment-covariate interactions using one and two-stage approaches to analysis. We use these findings, together with those of others and theoretical underpinnings, to explicitly consider the tradeoffs between computational and statistical complexity with the ability to minimise potential bias and provide insights into treatment effectiveness. Our aim is to provide pragmatic guidance on choice of methods.

**Table 1:** Key characteristics of one-stage models in approximate order of increasing computational complexity.

	Treatment effect	Treatment-covariate interaction	Within and across trial coefficients
Model 1	fixed	NA	NA
Model 2	random	NA	NA
Model 3	random	fixed	NA
Model 4	random	fixed	NA
Model 5	random	fixed	yes
Model 6	random	random	NA

Models 3 to 5 have fixed treatment-covariate interactions such that the effect of the covariate and the treatment-covariate interaction are common to all trials.

## Methods

The dataset comprises IPD collected as part of an international collaborative IPD meta-analysis evaluating antiplatelet agents for the prevention of pre-eclampsia [29] in pregnancy. We explored potential treatment interactions by previous “high risk” pregnancy, history of hypertension in pregnancy, previous infant small for gestational age, maternal renal disease, diabetes, and hypertension (categorical covariates) and maternal age and gestational age at randomisation (continuous covariates).

**Figure 1:**
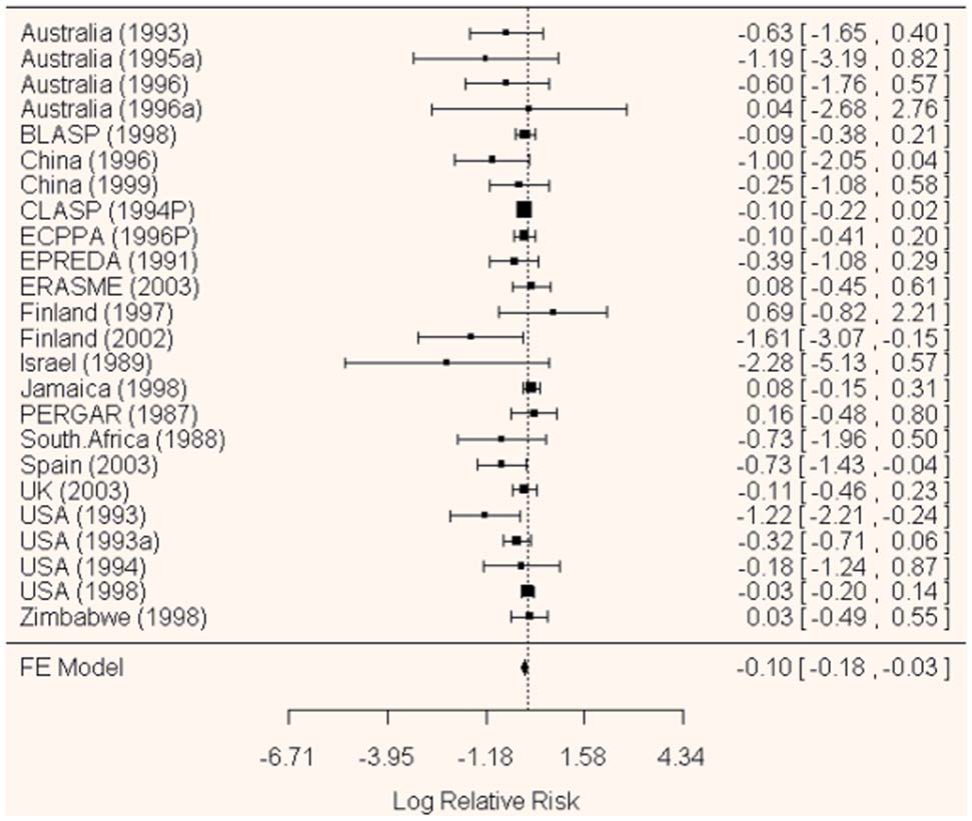
Forest plot of relative risks of developing pre-eclampsia (fixed-effect inverse variance model based on two-stage analysis replicating the analysis of [**29**]). Q(df = 23) = 31.19, p = 0.12, I^2^ = 26.3.

**Table 2:** Estimates of relative risk and heterogeneity (Tau^2^).

	Relative risk	95% CI	Amount ofHeterogeneity
Two-stage fixed	0.90	0.83 to 0.96	NA
One-stage fixed	0.90	0.83 to 0.97	NA
Two-stage random	0.87	0.78 to 0.97	0.011 (se 0.016)
One-stage random	0.90	0.83 to 0.97	0 (se 0.000)

### The Overall Treatment Effect

The previously published two-stage meta-analysis [29] was replicated. This analysis presented data on maternal pre-eclampsia from 24 randomised controlled trials, with a total of 30, 822 women. An equivalent one-stage fixed-effect model was fitted using logistic regression (model 1, see supporting information S2 for full model specifications). A two-stage random-effects analysis [30] was performed and compared to its one-stage random-effects equivalent, a random-effects logistic regression model (model 2, supporting information S2).

**Table 3:** Relative risks and p values for the interaction between treatment and categorical covariates using one and two-stage models.

		Two-stage		One-stage	
Subgroup	Category	Relative risk(95% CI)	Interactionp value	Relative risk(95% CI)	Interaction coefficient (standard error) p value
First pregnancy with/withouthigh risk factor	with	0.90 (0.76 to 1.08)	0.71	0.88 (0.66 to 1.09)	0.03 (0.13) p = 0.81
	without	0.87 (0.75 to 1.02)		1.16 (1.00 to 1.31)	
Second pregnancy with/withouthigh risk factor	with	0.89 (0.81 to 0.99)	0.56	0.88 (0.78 to 0.98)	−0.08 (0.17) p = 0.62
	without	0.98 (0.73 to 1.33)		0.95 (0.63 to 1.27)	
Second pregnancy with/withoutHistory of hypertension	Yes	0.86 (0.77 to 0.97)	0.25	0.88 (0.49 to 1.25)	−0.07 (0.10) p = 0.46
	No	0.96 (0.82 to 1.12)		0.94 (0.53 to 1.35)	
Renal disease	Yes	0.63 (0.38 to 1.06)	0.23	0.60 (0.35 to 1.04)	−0.43 (0.31) p = 0.17
	No	0.90 (0.82 to 0.96)		0.90 (0.82 to 0.98)	
Diabetes	Yes	0.63 (0.38 to 1.06)	0.26	0.71 (0.35 to 1.06)	−0.21 (0.19) p = 0.27
	No	0.90 (0.82 to 0.96)		090 (0.81 to 0.98)	
Hypertension	Yes	0.97 (0.84 to 1.12)	0.28	0.97 (0.82 to 1.15)	0.10 (0.10) p = 0.32
	No	0.88 (0.81 to 0.96)		0.89 (0.82 to 0.96)	
Previous small for gestationalage infant	Yes	1.05 (0.86 to 1.28)		1.05 (0.80 to 1.36)	
	No	0.85 (0.73 to 0.98)	0.27	0.85 (0.69 to 1.05)	0.25 (0.14) p = 0.07
	No previous infant	0.89 (0.79 to 0.99)		0.85 (0.75 to 1.32)	

The two-stage model with fixed-effect replicating the analysis of [29]. One-stage models were consistent whether treatment effects were fixed or random.

### Treatment-covariate Interactions

Two-stage analyses to investigate the association between treatment effect and covariates, that is, treatment-covariate interactions, were conducted by subgroup analysis for each covariates of interest. It was not possible to build a one-stage multivariate model incorporating all covariates, because different subgroups were reported in different trials, thus each covariate was considered in turn. One-stage fixed and random-effects analyses were conducted by extending regression models 1 (fixed) and 2 (random) to include the covariate and an interaction term between treatment and covariate as proposed by [8], (model 3, table 1).

**Table 4:** Relative risk and p value for treatment-covariate interactions for continuous covariates.

Subgroup	Category	Two-stage		Continuous covariate coefficients	One-stage	
		Relative risk(95% CI)	Interaction test p- value		Relative risk, *Odds ratio(95% CI)	Interaction coefficient p- value
Maternal age (years)	<20	0.97 (0.78 to 1.20)		Treatment	0.88 (0.87 to 0.89)*	
	20–35	0.87 (0.80 to 0.95)				
	>35	1.02 (0.83 to 1.26)	0.35	Interaction	1.00 (0.98 to 1.01)*	0.85
Gestational age at randomisation (weeks)	<20	0.87 (0.79 to 0.96)		Treatment	0.90 (0.84 to 0.95)	
	≥20	0.95 (0.85 to 1.06)	0.2	Interaction	1.00 (0.99 to 1.01)	0.53

We used presence of a high risk factor (as defined by [29], including hypertension or history of hypertension, renal disease and diabetes) as a dichotomous covariate, and maternal age as a continuous covariate, in further analysis of treatment-covariate interaction using more complex one-stage models (table 1). The two-stage approach considered three age categories (<20 years, 20 to 35 years, >35 years).The risk of pre-eclampsia increases for women over 35 years old [31]. However, the relationship between age and risk of pre-eclampsia may not be linear. Therefore, in addition to standard models assuming a linear relationship between age and risk of pre-eclampsia, one-stage models were constructed using quadratic terms to allow elevated risk in woman above and below the median age of 34.

**Table 5:** Comparison of one-stage models including interaction between antiplatelets and presence of a high-risk factor.

Model	Treatment coefficient		Interaction coefficient		AIC
	Log odds ratio (se)	p	Log odds ratio (se)	p	
3	−0.13 (0.07)	0.08	0.006 (0.09)	0.94	16189
4	−0.12 (0.07)	0.09	0.007 (0.09)	0.93	16137
5	−0.16 (0.06)	0.01	0.001 (0.006)	0.85	16200
6	−0.13 (0.07)	0.08	0.006 (0.09)	0.94	16199

**Table 6:** Comparison of one-stage models including interaction between antiplatelets and maternal age.

Model	Treatment coefficient		Interaction coefficient		AIC
	Log odds ratio (se)	p	Log odds ratio (se)	p	
3	−0.12 (0.004)	0.005	0.001 (0.006)	0.85	16199
4	−0.12 (0.004)	0.005	0.001 (0.006)	0.79	16190
5	−0.10 (0.05)	0.04	0.0004 (0.007)	0.94	13998
6	−0.15 (0.04)	0.0007	−0.001 (0.008)	0.86	16200

A range of models making different assumptions were used to analyse interactions between treatment and high risk factor and between treatment and maternal age. Model 3 assumes that the effect of the covariate and the treatment-covariate interaction are common to all trials. Model 4, however, allows for independent effects of the covariate across trials. Model 5 separates the within-trial information on the treatment-covariate interaction from the across-trials information. A final novel one-stage-model incorporates random-effects for both the treatment effect and the treatment-covariate interaction (model 6). We also explored the ease with which one-stage models could be extended to include multiple covariates or treatment-covariate interactions.

**Table 7:** Tradeoffs between analytical, computational and statistical complexity, ability to minimise potential bias and provide insights into treatment-covariate interactions.

Method	Computational and statistical complexity	Potential problems
Two-stage subgroup analysis	***Low:*** Requires only standard meta-analysis techniques and interaction tests.Available in several meta-analysis packages (eg. Cochrane Review Managerwhich requires pre-processing of IPD analyses within trials and SHARRP).Possible in most statistical packages (e.g. R, Stata).	***High:*** Limited statistical power. Potential for aggregation bias if trials lack data in some subgroup categories.
Two-stage, combining within-trial regression coefficients [9], [19]	***Moderate:*** Requires regression models estimating treatment effect and treatment-covariate interaction in each trial, and meta-analysis. Possible in statisticalpackages with regression and meta-analysis facilities (R, Stata).	***Low:*** Intermediate statistical power. Eliminates potential aggregation bias.
Simple one-stage regression [8]	***Moderate to high:*** Requires some experience in fitting regression models.Possible in R, Stata, SAS or equivalent.	***Moderate:*** Maximal statistical power. Potential for aggregation bias.
Complex one-stage regression (e.g. separating within- and across-trial information[7], [9]	***High:*** Requires expertise in fitting mixed-effect regression models andprogramming ability in R, Stata, SAS or equivalent. May require specialistsoftware such as WinBUGS. Statistical support is recommended.	***Low:*** Intermediate to high statistical power. Eliminates aggregation bias if only within-trials information considered.

**Table 8:** Pragmatic guidance for IPD systematic reviews and meta-analyses of intervention effects based on randomised trials.

1.	Estimate overall intervention effects and generate forest plots using conventional two-stage methods.
2.	Fit a two-stage analysis combining within-trial regression coefficients, to eliminate aggregation bias. Forest plots of interaction coefficients from such analyses are particularly useful for graphical display.
3.	If statistical support is available fit simple one-stage models with single treatment-covariate interactions (model 3). Compare with two-stage results.
4.	If possible and statistical support is available, fit one-stage models separating within and across trials information (model 5 or 6). Is there evidence of aggregation bias? Do within- and across-trials results differ?
	○ If there is evidence of aggregation bias: Report results from the within-trials association from model 5 or 6; or within-trial regressions where one- stage analysis was not possible.
	○ If there is no evidence of aggregation bias: Report results from model 3, if similar to model 5 or 6. These results are likely to have greater precision than two-stage analysis results.
5.	If statistical support is available, consider extending model 3 (in the absence of aggregation bias) or models 5 or 6 (with aggregation bias) to include multiple covariates and interactions. Compare multiple models, select a best fitting model and report its results, with a summary of all models considered.

Two-stage analyses were undertaken using the Metafor package [32] in R (2.14.1). One-stage models were fitted via the Lme4 package. The code to fit the one-stage models is included alongside the full model specifications in the supporting information S2.

## Results

The overall estimates of effect of anti-platelets in preventing pre-eclampsia obtained by one and two-stage approaches were compared (table 2). One and two-stage fixed-effect estimates were identical. There were minor differences between the models where random-effects were included because of the different estimates of heterogeneity (total variation between between studies). One-stage models estimated zero heterogeneity (Tau^2^) indicating no variation between studies. The standard (method-of-moments) estimate of heterogeneity in the two-stage approach was 0.011 (Q = 28.98, p = 0.18, I^2^ = 21%) indicating minimal variation, but both restricted maximum likelihood and maximum likelihood approaches estimated heterogeneity as zero (I^2^ = 0.01%). Heterogeneity therefore appears sensitive to method of computation, but not to choice of one or two-stage model directly. However, none of these differences were of material importance or would lead to different clinical interpretation of findings. The forest plot (figure 1) illustrates the minimal heterogeneity with associated statistics from the two-stage fixed-effect model.

### Comparison of Treatment-covariate Interaction Estimates from One and Two-stage Models

Neither one-stage nor two-stage methods identified any statistically significant interactions between the effect of anti-platelet administration and any of the types of women considered. There are important differences in the way results are presented between the approaches. Two-stage analyses generally present a p-value for an interaction test, whilst one-stage models provide interaction coefficients. Generally, in the two-stage approach effect sizes are only presented for subgroup categories (e.g. separately for men and for women) when there is clear evidence of a differential effect of the intervention, as indicated by statistically significant test for interaction. In which case the clinically utility of the intervention for each type of participant group is likely to be best judged with respect to the particular effect estimate obtained for that group (e.g. the effect estimate obtained for men will be used to make decisions about the use of interventions in men). Where there is no indication that particular types of participant benefit disproportionally from the intervention, as indicated by a non significant test for interaction, the overall result generally remains the best estimate to use when making clinical judgements of utility (e.g. the same overall effect will be used to make decisions about the treatment of both men and women). One-stage models generally present regression and interaction coefficients. When there is evidence of a differential effect of the intervention (e.g. women benefit more than men) the coefficients can be converted to effects to aid clinical interpretation. Here we present effects for all subgroups to facilitate comparison of one and two-stage model output along with p-values from associated tests of interaction. In actuality, neither approach would generally present results separately by subgroup category as there were no indications of differential effectiveness. There were no consistent differences between one and two-stage methods in terms of the size, precision or differences between subgroups (table 3).

There were some numerical differences in results for continuous covariates, although the differences were not clinically significant (table 4). The estimates of effect were very similar, but the p values for interaction were larger in the one-stage models, reflecting the tight confidence intervals around the treatment-covariate interaction term. This coupled with the interaction estimates indicating no effect, increases certainty about the lack of interaction in comparison to two-stage models (table 4).

One-stage models have the advantage of avoiding potentially arbitrary dichotomisation of continuous covariates and allow exploration of non-linear relationships. The inclusion of quadratic terms in models with maternal age did not substantively alter the treatment or treatment-age interaction coefficients. Model comparison indicated that simpler models, treating maternal age as linear, represented a better trade-off between the amount of variation explained and complexity than did models with quadratic terms. One-stage models did not consistently converge (models crashed) when estimating relative risks, but did converge when outcomes were measured as odds ratios. This reflects the additional complexity of modelling relative risk (which requires inclusion of a link function: see supporting information S2) in comparison to odds ratios which are a natural output of logistic regression models. Further comparison of one-stage models was therefore based on odds ratios.

### Comparisons of One-stage Models

The full range of one-stage models described in the introduction (table 1) were compared exploring treatment-covariate interactions with a dichotomous (table 5) and continuous covariate (table 6). Estimates of interaction and associated standard error were generally consistent across models. Model 5 which separated within- and across-trial covariate treatment interactions had the smallest estimate of within-trial interaction in both cases. This suggests that, in these instances, aggregation bias is resulting in over-estimates of treatment-covariate interaction. All models, however, clearly demonstrate that there is no evidence of interaction between treatment and any of the covariates investigated.

The additional complexity of model 5 was warranted in terms of the extra variability the model explained in comparison to other models where maternal age was concerned. Model fit, measured in terms of AIC was similar across models except for model 5, which had a substantially lower AIC than the other models in the analysis of maternal age, suggesting a better model fit, and that accounting for aggregation bias was important in that analysis (table 6).

The one-stage models performed similarly in terms of robustness and speed of convergence (measured in seconds rather than minutes), although convergence of the models was not always possible when calculating relative risks rather than odds ratios. Exploratory analyses based on inclusion of multiple covariates simultaneously suggest that extending the models (particularly model 5) beyond a single treatment-covariate interaction may not always be possible as multivariate models were prone to crash.

## Discussion

The selection of analytical method for IPD may not be straightforward. Advocates of a one-stage approach point to increased power to detect treatment-covariate interactions, ability to control for aggregation bias and also suggest that one-stage approaches may provide deeper insights into the data by allowing testing of different assumptions about model structure and adjustment for multiple covariates [9], [19], [27], [28]. However, these potential advantages come at the cost of computational complexity and require additional statistical expertise in comparison to the two-stage approaches used in most IPD analyses. Advocates of a two-stage approach question whether these theoretical benefits are realised in practice and whether they lead to differing clinical conclusions. This analysis of a large data set with a dichotomous primary outcome was consistent with previous analyses of smaller data sets with time-to-event outcomes [27], [28] strengthening the view that one and two-stage approaches will often produce similar results in practice. Clearly, this represents a limited body of empirical evidence, but it does indicate that those considering undertaking an IPD analysis should not necessarily be deterred by a perceived need for sophisticated statistical methods, irrespective of the type of outcome.

In this example, the increased power of one-stage methods was not manifest in tighter confidence intervals for overall treatment effects or for treatment-covariate interactions of the seven categorical covariates investigated. However, when using a one-stage approach, the treatment-covariate interaction terms were more precisely estimated for continuous covariates. The lack of interaction may be more apparent in one-stage models which display interaction coefficients and standard errors than two-stage models where interactions are assessed using p- values from subgroup analysis. This may be of importance where p-values are close to statistically significant boundaries.

One-stage models have the advantages of not requiring the potentially arbitrary dissection of covariates, and ability to test for non-linear relationships for continuous covariates. However, interpretation of the resulting coefficients may be difficult. Centering treatment effects on median values is appropriate where there are no interactions as this reflects the population for whom the estimate is most applicable. Where interactions are identified, it may be appropriate to express coefficients in terms of treatment effects for categorical groups identified in protocols, as is standard in presentation of results from two-stage analysis.

Aggregation bias is a potential problem in any meta-analysis. Avoiding this bias by using IPD and distinguishing between within-trials and across-trials information is therefore important. To eliminate such bias, only within-trial information on the association between treatment effects and covariates should be considered. This is possible in both one-stage (see model 5) and two-stage approaches. In a two–stage analysis aggregation bias can be avoided by only including trials that report effects for all subgroup categories. Alternatively, within-trial treatment-covariate interactions can be identified by undertaking regression analyses within each trial and combining regression coefficients in a meta-analysis across trials. One-stage models which separate within and across trial treatment-covariate interactions provide direct measures of effect and precision thereby allowing quantification of the effects of aggregation bias. Model specification, can allow statements to be made directly about the magnitude and significance of aggregation bias (supporting information S2).

A key advantage of a one-stage approach is the flexibility in terms of the models that may be fitted. One-stage models allow for the inclusion of multiple covariates in a single model, multiple random-effects on different parameters and the separation of within and across-trials information. The different models may not necessarily lead to different results, as was found in this analysis. One-stage models may also be compared, in terms of both goodness of fit and parsimony of model, by using, for example, the AIC statistic. This allows the selection of a “best fitting” model to be identified across a range of possible models. Use of AIC reduces the risks of over-fitting and data-dredging by including too many, irrelevant covariates or specifying multiple implausible models. The rationale for choice of model should be transparently reported and justified to ensure that the flexibility of the one-stage approach does not result in selective reporting of results. As with any meta-analysis, *a priori* identification of covariates and clinically meaningful combinations of covariates in a protocol (e.g. [33]) is essential.

Whilst two-stage approaches may sometimes be unrealistically simple, one-stage approaches may be intractably complex. In this analysis models expressed in terms of relative risk could not always be applied, and problems arose as multiple covariates were included in any model. Simpler models may be preferable, with fewer covariates, fewer random-effects and expressing outcomes as log odds rather than log relative risk. Alternatively, use of software such as WinBUGS [34], may be required to allow a simulation approach to analysis. The costs associated with the former strategies relate to the realism of the simplifying assumptions and generalisability of results whilst use of alternative software may require additional statistical expertise.

One- stage models have greater statistical complexity and are therefore harder to interpret. Statistical support is an important pre-requisite for the implementation of these models. Careful interpretation and explanation of the coefficients is required. For dichotomous outcomes expression of results as odds ratios or risk ratios with 95% confidence intervals is preferable to display of raw coefficients as this is more meaningful to most non statisticians. Reporting guidelines have not yet been developed specifically for IPD analyses but should clearly consider the reporting of one-stage models with emphasis on the explicit value judgements regarding model structure, sensitivity of results to model choice, and interpretation of regression coefficients.

### Recommendations for Analysis of IPD Systematic Reviews

Here we suggest a pragmatic approach to analysis in IPD reviews based on existing empirical comparisons of one and two-stage methods (the current work, [9], [27], [28]), theory [16], [19], and simulation studies [15] as well as personal judgements about the tradeoffs between computational and statistical complexity and the potential for bias associated with different methods and types of data.

Irrespective of the final approach, performing a two-stage analysis as an initial step is generally advisable. This generates forest plots enabling results across trials to be compared visually, heterogeneity investigated and differences across subgroups visualised, all of which are essential in understanding the dataset underlying the review. We suggest that for reviews of large randomised trials that are homogeneous in populations and design, a two-stage analysis will often be sufficient. Large numbers of participants mean that lack of statistical power is unlikely to be an issue and clinical homogeneity of trials reduces the risk of aggregation bias. In such situations two-stage and one-stage methods are likely to give similar results. However, a one-stage analysis may still be preferred for evaluating treatment-covariate interactions of continuous covariates, to avoid arbitrary categorisation and to incorporate non-linear relationships. They may also be of use for fitting single models including multiple covariates, particularly where covariates are expected to be highly correlated.

One-stage methods may be most appropriate when trials are small, participant numbers are few and where there is clinical heterogeneity across trials. In this case two-stage methods may lack statistical power and subgroup analyses may be affected by aggregation bias, particularly if some trials did not include participants in some specified subgroup categories.

To avoid aggregation bias in two-stage analyses, models should be fitted to estimate treatment-covariate interactions within each trial, and these estimates pooled across trials, rather than using conventional subgroup analysis. Where possible, one-stage models should be parameterised to separate within and across trial treatment-covariate interaction, at least as a sensitivity analysis. Where one-stage models are used a range of plausible models should be fitted (ranging from too simple to too complex) and these models compared.

Trade-offs between computational and statistical complexity and potential for problems such as aggregation bias and lack of statistical power are explicitly considered in table 7 with guidance on methodology for routine application summarised in table 8. More sophisticated methods are likely to be required for analysis of non-randomised data particularly if adjustment for multiple confounders is required.

Major benefits of obtaining IPD are accrued prior to analysis and where an IPD review evaluates effectiveness based on sufficient data from randomised controlled trials, one-stage statistical analyses may not add much value to simpler two-stage approaches. Researchers should therefore not be discouraged from undertaking IPD synthesis through lack of advanced statistical support.

## Supporting Information

Figure S1 **Graphical representation of the type of aggregation bias presumed to be most prevalent in medical data-sets.**
(TIF)

Supporting Information S1 **Aggregation bias.**
(DOCX)

Supporting Information S2 **Full model specifications and R code for implementation.**
(DOCX)
