# Supplementary material for: Statistical Analysis of Individual Participant Data Meta-Analyses: A Comparison of Methods and Recommendations for Practice
Source: PLoS One. 2012 Oct 3;7(10):e46042. doi: 10.1371/journal.pone.0046042 (PMC3463584; doi:10.1371/journal.pone.0046042)
Supplement: Supporting Information S1 — Aggregation bias. (DOCX) [file pone.0046042.s002.docx]

**Supporting Information S1**

Aggregation bias (also called ecological bias or ecological fallacy) is the erroneous interpretation of data where inferences about the nature of individuals are based solely on aggregate statistics collected for the group to which those individuals belong.

An early example was identified in sociology where an aggregate analysis illustrated that US states with the highest proportions of foreign born population also had the highest literacy rates, even though the foreign born had lower literacy rates than the native born population [18]. Here the averages were potentially misleading because immigration was higher in the more prosperous and literate states. Robinson [18] cautioned against deducing conclusions about individuals on the basis of population-level, or ecological data, hence use of the term ecological fallacy.

There has been no systematic study of aggregation bias in medicine, but Berlin *et.al.* [17] provide a concrete example based on the effectiveness of anti-lymphocyte antibody induction therapy among renal transplant patients. Aggregate level analysis did not identify any subgroups of patients whom benefited from treatment more than others but the patient-level analysis found that treatment was significantly more effective among patients with elevated panel reactive antibodies (PRA) than among patients without elevated PRA (Berlin *et.al.* 2002).

The general lack of treatment covariate interactions in IPD analyses in comparison to meta-regressions suggest that more commonly aggregation bias in medicine is likely to be manifest as no or very weak relationships between treatment and a covariate within trials but stronger relationships across trials. We have illustrated this with a hypothetical outcome and treatment in figure S1.

There are clearly no relationships between the arbitrary treatment effectiveness outcome and covariate within each of the four trials. However, because the mean value of the covariate is different in each trial aggregate level analysis suggests that there is a positive relationship between treatment and covariate as indicated by the meta-regression line.

In addition to differential identification of an effect, it is even possible for the differences between studies to display the opposite pattern to that observed within each study. For these reasons IPD analyses are better suited to identifying variation in treatment effects with patient level covariates than aggregate analyses especially where they separate within and between study treatment covariate interactions.
