# Supplementary material for: Statistical Analysis of Individual Participant Data Meta-Analyses: A Comparison of Methods and Recommendations for Practice
Source: PLoS One. 2012 Oct 3;7(10):e46042. doi: 10.1371/journal.pone.0046042 (PMC3463584; doi:10.1371/journal.pone.0046042)
Supplement: Supporting Information S2 — Full model specifications and R code for implementation. (DOCX) [file pone.0046042.s003.docx]

**Supporting Information S2**

***Model 1***

*One-stage fixed-effect model*

$$logit\left( p_{ij} \right)=log\left( \frac{p_{ij}}{1-p_{ij}} \right)=\alpha_{i}+\beta x_{ij}$$

Where p_ij_ is the probability of an event for person j in trial i, x_ij_ denotes the treatment received, α_i_ is the baseline risk in each trial and β_i_ is the log odds ratio of the treatment effect. To obtain the log relative risk, replace the logit link with a log link function.

*R code:* glm(event~trial+treat, family=binomial)

*Or for relative risk:* glm(event~trial+treat, family=binomial(link=”log”))

***Model 2***

*One-stage random-effects model*

$$\begin{matrix} logit\left( p_{ij} \right)=\alpha_{i}+\beta_{i}x_{ij} \\ \beta_{i}\sim N\left( \theta,\tau^{2} \right) \end{matrix}$$

Here treatment effect varies across trials, distributed normally around the overall effect θ, with heterogeneity τ^2^.

*R code:* glmer(event~trial+treat+(treatn-1|trial), family= binomial)

** treatn is a numerical coding for treatment (Experimental=1, Control=0)*

***Model 3***

*One-stage random-effects model with treatment-covariate interaction (Turner et.al. (2000))*

$$\begin{matrix} logit\left( p_{ij} \right)=\alpha_{i}+\beta_{i}x_{ij}+\gamma z_{ij}+{\delta x}_{ij}z_{ij} \\ \beta_{i}\sim N\left( \theta,\tau^{2} \right) \end{matrix}$$

Where z_ij_ is the coding for the covariate, γ is the baseline effect of the covariate and δ the treatment-covariate interaction.

*R code:* glmer(event~trial+treat*covar+(treatn-1|trial), family= binomial)

***Model 4***

*One-stage random-effects model with treatment-covariate interaction (Simmonds (2005))*

$$\begin{matrix} logit\left( p_{ij} \right)=\alpha_{i}+\beta_{i}x_{ij}+\gamma_{i}z_{ij}+{\delta x}_{ij}z_{ij} \\ \beta_{i}\sim N\left( \theta,\tau^{2} \right) \end{matrix}$$

This model allows for independent effects of the covariate on risk across trials ($\gamma_{i}$ rather than the $\gamma$ of Turner *et.al.*).

*R code:* glmer(event~trial*covar+treat*covar+(treatn-1|trial), family= binomial)

***Model 5***

*One-stage random-effects model allowing for aggregation bias (Simmonds-Riley).*

$$\begin{matrix} logit\left( p_{ij} \right)=\alpha_{i}+\beta_{i}x_{ij}+\gamma_{i}z_{ij}+{\xi x}_{ij}{(z}_{ij}-\bar{z_{i}})+\eta x_{ij}\bar{z_{i}} \\ \beta_{i}\sim N\left( \theta,\tau^{2} \right) \end{matrix}$$

Where $\bar{z}_{i}$ is the mean of the covariate within-trial i, ξ is the desired within-trial interaction and $\eta$ is the across-trials interaction. This model may be expressed as:

$$\begin{matrix} logit\left( p_{ij} \right)=\alpha_{i}+\beta_{i}x_{ij}+\gamma_{i}z_{ij}+{\delta x}_{ij}z_{ij}+\kappa x_{ij}\bar{z_{i}} \\ \beta_{i}\sim N\left( \theta,\tau^{2} \right) \end{matrix}$$

Where $\delta=\xi$ and$\kappa=\eta-\xi$, thus κ is a measurement of aggregation bias.

*R code:* glmer(event~trial*covar+treat*covar+intermean+(treatn-1|trial),family=binomial)

** Where* intermean *is a new variable being zero in the control group and equal to the trial-level covariate mean in the experimental group (i.e treatn*mean covariate).*

***Model 6***

*One-stage model with random treatment and interaction effects*

$$\begin{matrix} logit\left( p_{ij} \right)=\alpha_{i}+\beta_{i}x_{ij}+\gamma_{i}z_{ij}+{\delta_{i}x}_{ij}z_{ij} \\ \left( \begin{matrix} \beta_{i} \\ \delta_{i} \end{matrix} \right)\sim N\left( \begin{matrix} \left( \begin{matrix} \theta\\ \delta\end{matrix} \right), & \left( \begin{matrix} \tau^{2} & c \\ c & \tau_{\delta}^{2} \end{matrix} \right) \end{matrix} \right) \end{matrix}$$

Where the interaction term δ has a random effect which may be correlated with the treatment random effect. Model 6 is a useful and novel alternative to model 5 for nominal covariate data (e.g. disease stage), where separation of within and across trial treatment interactions is not possible, as the additional random effect term minimises the impact of aggregation bias.

*R code:* glmer(event~trial*covar+treat*covar+(treatn+treatn:covar-1|trial), family= binomial)
